# *Ficus religiosa* leaf extract mitigates the neurofibrillary tangles and amyloid plaques in aluminium chloride exposed Wistar rat brain

**DOI:** 10.1007/s13205-025-04647-1

**Published:** 2026-01-05

**Authors:** Amit Massand, Rajalakshmi Rai, Ashwin Rohan Rai, Teresa Joy

**Affiliations:** 1 Department of Anatomy, PES Institute of Medical Sciences and Research, PES University, Electronic city, Bangalore, India; 2 Department of Anatomy, Kasturba Medical College Mangalore, Manipal Academy of Higher Education, Manipal, India; 3 Department of Anatomy, American University of Antigua College of Medicine, University Park, Jabberwock Beach Road, Coolidge, Antigua Antigua and Barbuda; 4 Department of Anatomy, Kasturba Medical College Mangalore, Manipal Academy of Higher Education, Manipal, India

**Keywords:** Aluminium, Ficus religiosa, Brain, Amyloid plaque, Neurofibrillary tangle, Locomotor activity

## Abstract

Aluminium (Al) deposition in different parts of the brain contributes significantly for the progression of neurodegenerative changes. The present study was undertaken to find out the influence of *Ficus religiosa* leaves against Al induced deposition of neurofibrillary tangles (NFT) and amyloid plaque in Wistar rats. We used rats of around 12-week age for the study. The animals were divided into 7 groups classified as control, Al, T200 & 300 as two extract treatment groups, FR200 & 300 which served as FR extract control and PRL, the prophylactic group. Aluminium exposure increased the expression of NFT and amyloid plaques along with decreased locomotor activity in the present study. However, these deleterious effects were improved in the form of reduced number of NFT and amyloid plaques by *Ficus religiosa* leaf extract treatment in a dose dependent manner. The outcomes of our study reveal the therapeutic potential of FR leaves against neurological disorders by combating the amyloid plaque and NFT formation.

## Introduction

Homeostasis of metals in our body plays an important role in wellbeing of an individual. However, if this homeostasis gets disturbed, it affects the structural integrity and function of nervous system and might give rise to neurological disorders. Excessive accumulation of heavy metals like aluminium (Al), mercury, iron, and lead are considered as risk factors for neurodegenerative disorders (Campbell 2006; Weisskopf et al. 2007). Tomljenovic & Shaw (2011) state that substantially high level of Al in the brain is associated with Alzheimer’ Disease, a well-known neurodegenerative disease.

In patients suffering from AD, amyloid plaques were observed in the brain along with increased level of aluminium, implying the connection between Al toxicity and AD (Miu & Benga 2006; Yumoto et al. 2009). Additionally, several investigations have clearly elucidated the correlation between AD and aluminium (Yumoto et al. 2009; Moore et al. 2000; Miu et al. 2003). Further studies also report that amyloid protein and tau protein aggregations increase in advanced cases of neurodegeneration (Iqbal et al. 2016; Burack et al. 2010) with impairment in cholinergic transmission leading to motor and cognitive impairment (Perez-Lloret & Barrantes 2016).

In modern era as the usage of Al compounds are considerably increasing, it can pose a major threat to the mankind, as per the reports of some studies on environmental poisoning (Robert & Yokel 2012; Jaishankar et al. 2014). Neurotoxic effect of Al compounds is caused mainly by the over production of free radicals and reactive oxygen species (Pasha & Oglu 2017). ROS result in cytotoxicity by disturbing the signalling pathway and as a consequence of this, neurotransmission is affected along with neuronal apoptosis and neurodegeneration (Sharma & Mishra 2006; Niu et al. 2005, Akinrinade et al. 2015). The causes of neurodegenerative diseases are multifactorial, therefore identifying different therapeutic strategies for mitigating neurodegeneration is the need of the hour. Also, development of effective medicine against the deleterious effects of heavy metals is the present need in todays polluted environment. Since last few years the plant derived therapeutic drugs are gaining popularity because of it being natural with less toxic effects. The therapeutic property of the plants can be contributed to its rich phytochemicals. Integrating plant-based medications with modern medicines might increase the effectiveness of treatment strategies for neurodegenerative diseases, like Alzheimer’s diseases (Tripathi et al., 2024).

*Ficus religiosa* (FR) is one such tree with several beneficial phytoconstituents (Chandrasekar et al. 2010). Popularly known as ‘bodhi tree’, it’s been widely used in traditional medicine for various illnesses. In India FR tree has got religious, mythological, and medicinal importance (Prasad et al. 2006). The leaves of FR were used as traditional medicine for epilepsy, asthma, diabetes, inflammatory diseases, and gastric disease (Panchawat 2012) with promising effect on Huntington’s rat model (Bhangale et al. 2016a) as well as for management of AD (Vinutha et al. 2007). Therefore, this study was undertaken to investigate the neuromodulatory role of FR leaf extract on amyloid plaques and neurofibrillary tangle formation in different areas of hippocampus and dentate gyrus, on motor function and expression of acetyl choline in AlCl^3^ administered *Wistar* rats.

## Materials and methods

### Animals

The present study utilized eighty-four male *Wistar* rats of about 12 weeks age, during the commencement of the study. The rats were maintained under the recommended room temperature, humidity, circadian rhythm of light and dark cycle as per ARRIVE guidelines. Three rats were housed in one polypropylene cage and had *ad libitum* access to rat chow which were obtained from Champaka Feeds and Foods, Bangalore, India. The study protocol was approved by institutional animal ethics committee of our university (KMC/MNG/IAEC/03–2020). The research was conducted in the central animal house of our institution from August 2021 to July 2022.

### Animal groups

The animals were divided into 7 groups with each group having 12 rats.

- Group 1 (control): Normal control where animals were fed with pellets and drinking water for 60 days duration without administering any drug.
- Group 2 (Al): Animals were administered with 100 mg/kg body weight (bw) of AlCl_3_ for 45 days. This is neurotoxicity induced group.
- Group 3 (T200): Animals were administered with 100 mg/kg bw of AlCl_3_ for 45 days, which was succeeded by 200 mg/kg bw of FR leaf extract for 15 days. This is treatment group with lower dose of FR leaf extract.
- Group 4 (T300): Animals were administered with 100 mg/kg bw of AlCl_3_ for 45 days, which was succeeded by 300 mg/kg bw of FR leaf extract for 15 days. This is treatment group with higher dose of FR leaf extract.
- Group 5 (FR200): Animals were administered with 200 mg/kg bw of FR extract alone for 15days.
- Group 6 (FR300): Animals were administered with 300 mg/kg bw of FR extract alone for 15days.
- Group 7 (prophylactic group- PRL): Animals were pre-treated with 200 mg/kg bw of FR leaf extract alone for 1 week followed by 100 mg/kg bw of AlCl3 and 200 mg/kg bw FR for 45days. This group is prophylactic group to check the preventive effect of FR leaf extract.

Sample size of our study was determined according to previous animal study on neurotoxicity (Sahu et al., 2013). Slides of tissues from different groups of rats were decoded to avoid manual bias while counting the neurons. Histopathological assessment of immunohistochemistry slides was performed with the help of blinded observer to avoid manual bias.

### Extraction of *Ficus religiosa* leaves

*Ficus religiosa* leaves were identified and collected from Shobhavan botanical garden, Moodbidri, Karnataka. The identity of the leaves was confirmed and certificate of identity of the FR leaves was issued by a well-known botanist Dr. HS Shenoy, Principal Scientist, Dr. Shivarama Karantha Pilikula Nisarga Dhama, Mangalore. The old, dried leaves were excluded, and medium ripe, green leaves were used throughout the study. As the amount of moisture in the atmosphere is more in this region, the leaves were allowed to dry in shade for over 6 months. When these leaves were dried completely, the coarse powder was made using the mixer grinder. Thus obtained coarse powder was put in the Soxhlet Apparatus along with ethanol (1:1with distilled water) to prepare the extract. Ethanol was used as a solvent in ratio of 1:1 along with distilled water.

### Tissue processing

Day after last administration of either AlCl^3^ or FR extract according to group, rats were deeply anesthetized by sodium pentobarbitol (45 mg/kg bw) with subsequent perfusion of 10% formalin. This was followed by brain removal after cranial cavity dissection, and it was stored in containers filled with 10% formalin for further fixation. After that paraffin blocks were prepared, serial sections (coronal sections) of brain (6–7 μm thick) with the hippocampus were taken using a rotatory microtome (Jung Biocutt 2035, Lieca Wetztar, Germany). These paraffin embedded tissue sections were studied with immunohistochemical stains containing primary and secondary antibody for Tau-protein and β-amyloid proteins.

### Estimation of β amyloid plaque formations

Tissue sections were mounted on poly-L-lysine coated dry slides, as described by Shi et al. (2012). After deparaffinization, followed by dehydration tissue was rinsed with distilled water. Then it was treated with 98–100% formic acid for three minutes for antigen retrieval, followed by washing it twice in PBS for five minutes. Then the sections were incubated in different solutions at following steps:

1. With 0.3% hydrogen peroxide for 10–15 min, to block the endogenous peroxidase and rinsed in PBS for two minutes.
2. With 2% normal horse serum in PBS for 20 min to stop non-specific binding of secondary immunoglobulin and rinsed in PBS for two minutes.
3. With diluted mouse anti-beta-amyloid primary antibody in PBS (1:100) and rinsed with PBS thrice for five minutes.
4. With secondary antibody biotinylated horse anti-mouse IgG diluted in PBS (1:400), for 30 min and rinsed thrice in PBS.
5. With HRP-streptavidin reagent diluted in PBS (1:400) to form Avidin Biotin Complex (ABC), for 30 min followed by rinsing in PBS thrice.
6. With a solution of diaminobenzidine (DAB) for 2–10 min and then rinsed in distilled water.

After the above process the tissues were dehydrated, cleared and mounted for viewing. For each hippocampal region (CA1 to CA4) 250 μm length area and for dentate gyrus 50µm^2^ area was taken and screening was performed using Nikon trinocular microscope (H600L). Finally, quantification of amyloid beta protein was performed with the help software NIS Elements Br version 4.30 as done by Madhyastha et al. (2002).

### Estimation of neurofibrillary tangle

After deparaffinization and dehydration tissue was rinsed with distilled water and NFT was evaluated according to the method of Fen et al. (2010). The tissue was warmed was for 30 min in 0.01 M citrate buffer (pH 6) in a water bath at 60˚C and then washed with distilled water (DW). Then the slide was placed in Phosphate Buffer (pH 7.4) for 5 min. Following this the slides were incubated in different solutions at following steps:

1. With 0.3% hydrogen peroxide for 20 min, to block the peroxidase and rinsed in PBS thrice for five minutes each.
2. With 5% Instant Calf Serum (ICS) for 60 min in 37˚C. The 5% ICS is prepared in PBS containing Triton X-100/Tween-20.
3. With primary antibody Polyclonal Rabbit Anti-human Tau that is diluted in PBS containing ICS (1:30,000), for overnight in room temperature and washed thrice in PBS for 5 min each.
4. With biotinylated Anti-goat IgG that is diluted in PBS (1:200), for 1 h at room temperature and washed thrice in PBS for 5 min each.
5. With Avidin Biotin Complex diluted in PBS (1:400) for 1 h. ABC solution was prepared by mixing reagent-B and reagent-A in PBS (1:50) and allowed to stand for 30 min before use. After incubation the tissue was washed thrice in PBS for 5 min each time.
6. Subsequently treated with a solution of 3,3´-diaminobenzidine (DAB) for 2–10 min to develop the colour and then rinsed PBS thrice for 5 min each.
7. The sections were dehydrated with ascending grades of alcohol, cleared with xylene and mounted for viewing.

After the above process the tissues were dehydrated, cleared and mounted for viewing. For each hippocampal region (CA1 to CA4) 250 μm length area and for dentate gyrus 50µm^2^ area was taken and screening was performed using Nikon trinocular microscope (H600L). Finally, quantification of NFT was performed with the help software NIS Elements Br version 4.30 as done by Madhyastha et al. (2002).

Neurofibrillary tangles and amyloid plaques were identified in different Cornua ammonis (CA) regions of hippocampus and dentate gyrus by a pathologist using Nikon trinocular microscope (H600L) and the images were clicked and uploaded to the computer. Finally, quantification of amyloid beta protein was performed with the help of software NIS Elements Br version 4.30 as described by Madhyastha et al. (2002). For each hippocampal region (CA1 to CA4) 250 μm length area and for dentate gyrus 50µm^2^ area was selected. The quantification of NFT and plaque was performed by multiple observers to avoid bias. As mentioned in the manuscript statistical analysis of the results was performed using One-way ANOVA and Tukey’s test for multiple comparison. The SPSS software was used for analysis and ‘p’ value less than 0.001 was considered highly significant. Values are expressed as Mean ± Standard Deviation (SD).

### Open field test

The locomotor and exploratory activity (emotional reactivity/anxiety and locomotor activity) of the rats were measured with the help of open field test as described by Knight et al. (2021).

Apparatus: A wooden box which is rectangular (100 × 100 × 40 cm) with the floor that consists of 25 equal squares (5 × 5 cm) and has 100 watts bulb fixed 60 cm above the centre, in the roof of the box, for illumination inside.

Rats and mice tend to avoid bright illuminated open spaces, so the open field environment acts as an anxiogenic stimulus and allows for measurement of anxiety induced locomotor activity and exploratory behaviours.

Procedure: Animals were allowed to explore the open field apparatus for 5 min before the evaluation. For the test each rat was placed in a corner of the box and the number of peripheral and central crossings entered by the rats in a-five-minutes duration was noted. The fraction of total exploratory time spent in peripheral area of sixteen squares (close to wall) and in the central area were measured for individual rat. Total time for exploration in each session was 5 min. Scoring was performed by recording the number of squares entered by the rat and the time spent by the rat in the square in that duration. Lesser time spent in the central nine squares reflects an avoidance of anxiogenic places. Number of peripheral squares crossed are indicators of exploratory behaviour.

### Estimation of acetylcholinesterase (AChE)

After anaesthetizing the animal with sodium pentobarbital (45 mg/kg bw) the brain was removed following cranial cavity dissection, transferred to normal saline and AChE expression was assessed by the method described by Ellman et al. (1961). Brain tissue homogenate was prepared with 0.1 M PBS of pH 7.4. The homogenized sample was centrifuged (10,000 x g) for 20 min at four degrees Celsius and a portion of the supernatant was taken and utilized for the AChE estimation.

AChE catalyzes the hydrolysis of acetylcholine to form choline & acetate and choline react with dithio p-nitrobenzoic acid (DTNB) to form 5-mercapto-nitrobenzoic acid (TNB). TNB has an absorption peak at 412 nm. The activity of AChE is calculated by measuring the increasing rate of absorbance at 412 nm. To each well of microplate containing 170 µL of reagent 3 working solution, 20 µL of sample was added. Then added 10 µL of reagent 4 working solution to each well and mixed completely for 5 s. With the help of microplate reader, the changes in absorbance at 412 nm was measured within 5 min. The OD value of 30 s and 330 s were recorded as A1 and A2, respectively. ΔA = A2-A1. Calculated according to the protein concentration of sample tissue.

### Statistical analysis

Statistical analysis of the results was performed using of One-way ANOVA and Tukey’s test for multiple comparison. The SPSS software was used for analysis and ‘p’ value less than 0.001 was considered highly significant. Values are expressed as Mean ± Standard Deviation (SD).

## Results

### Amyloid plaques: (Figures 1, 2, 3, 4 and 5; Table 1)

The amyloid- plaques were found in all areas of the hippocampus (CA1, CA2, CA3, CA4) and dentate gyrus in Al group. In control group, plaques were found in CA1, CA4 and dentate gyrus only in minimal number. In aluminium intoxicated rats the number of plaques increased substantially (*p* < 0.001) in all regions of hippocampus including dentate gyrus when compared with control group (Fig. 6). The treatment group T200 has shown decline in amyloid plaques in all areas of hippocampus and dentate gyrus compared to Al group. Whereas the T300 group rats did not show any amyloid plaques in CA2 & CA3 regions and in CA1 & CA4 regions it declined significantly (*p* < 0.001). In prophylactic group the amyloid plaques were only seen in dentate gyrus and were nil in all regions of hippocampus, thus showing significant improvement (*p* < 0.001) compared with Al group.

**Fig. 1:**
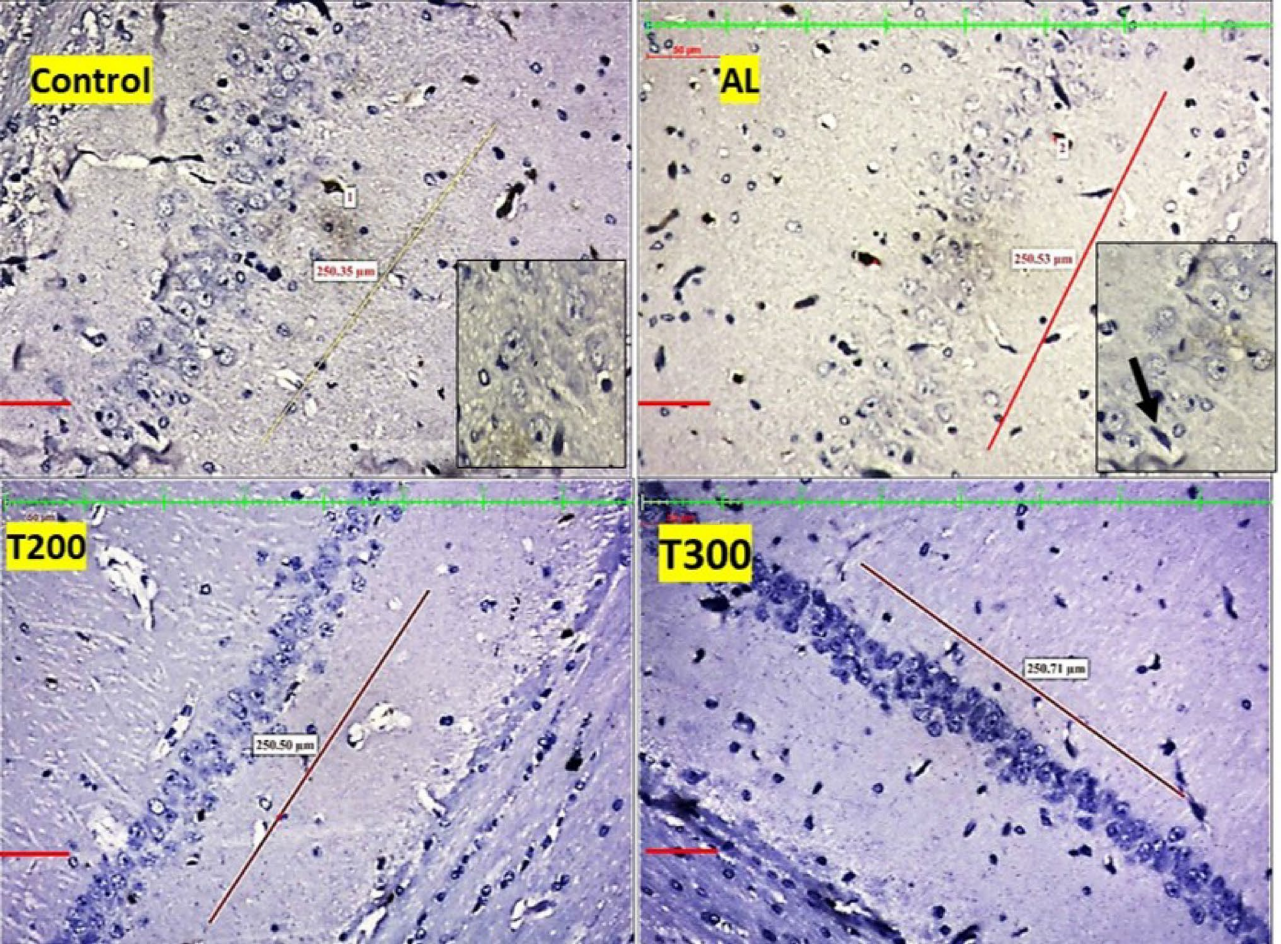
Photomicrograph of CA1 area of hippocampus of rat brain tissue immunostained with anti- β-Amyloid antibody for β-Amyloid plaques (BAP). BAP appears like dark colored aggregation, usually circular extracellular deposits (black arrow); Scale bar-50µ

**Fig. 2:**
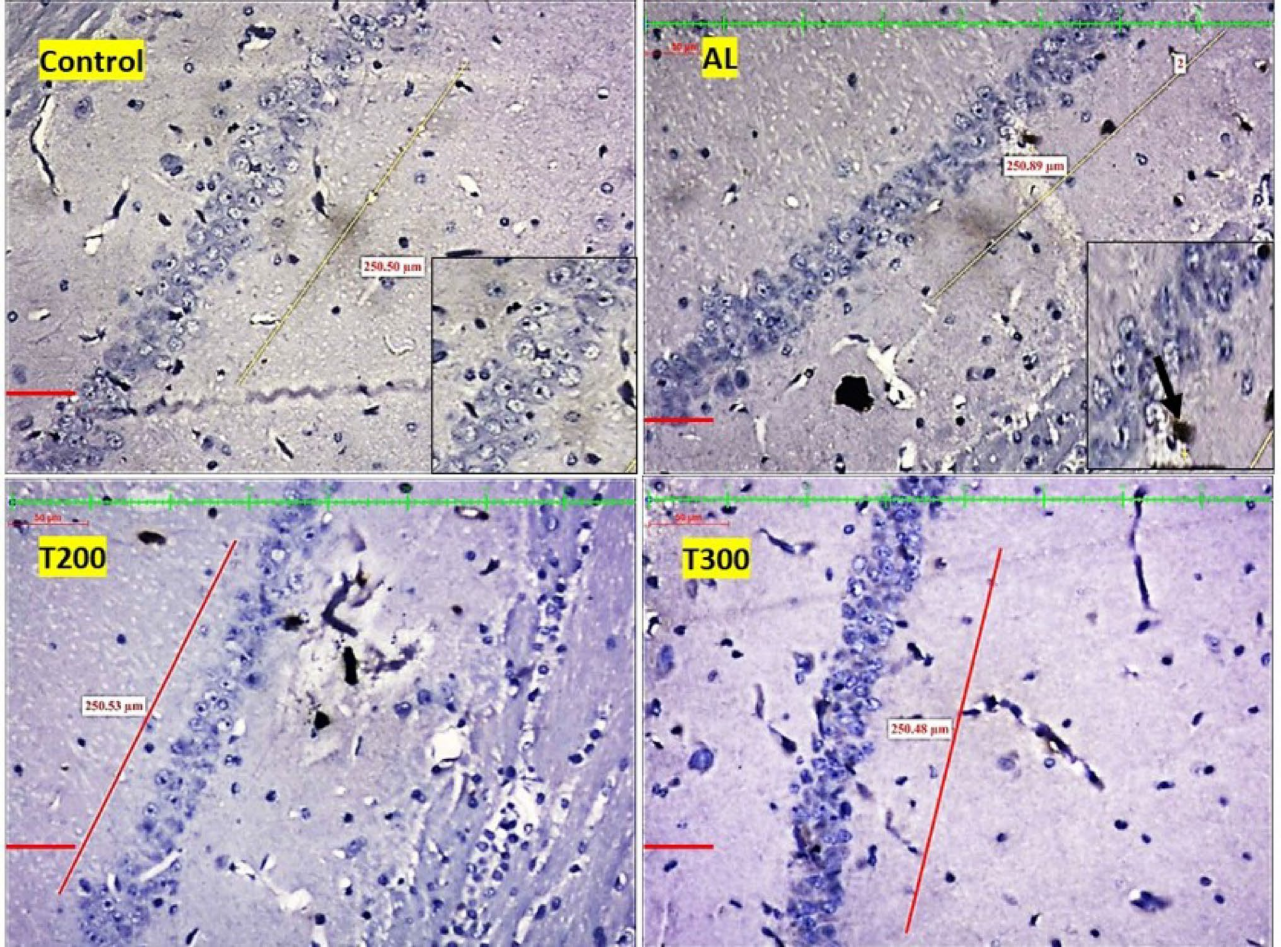
Photomicrograph of CA2 area of Hippocampus of rat brain tissue immunostained with anti- β-Amyloid antibody for β-Amyloid plaques (BAP). BAP appears like dark colored aggregation, usually circular extracellular deposits (black arrow); Scale bar-50µ

**Fig. 3:**
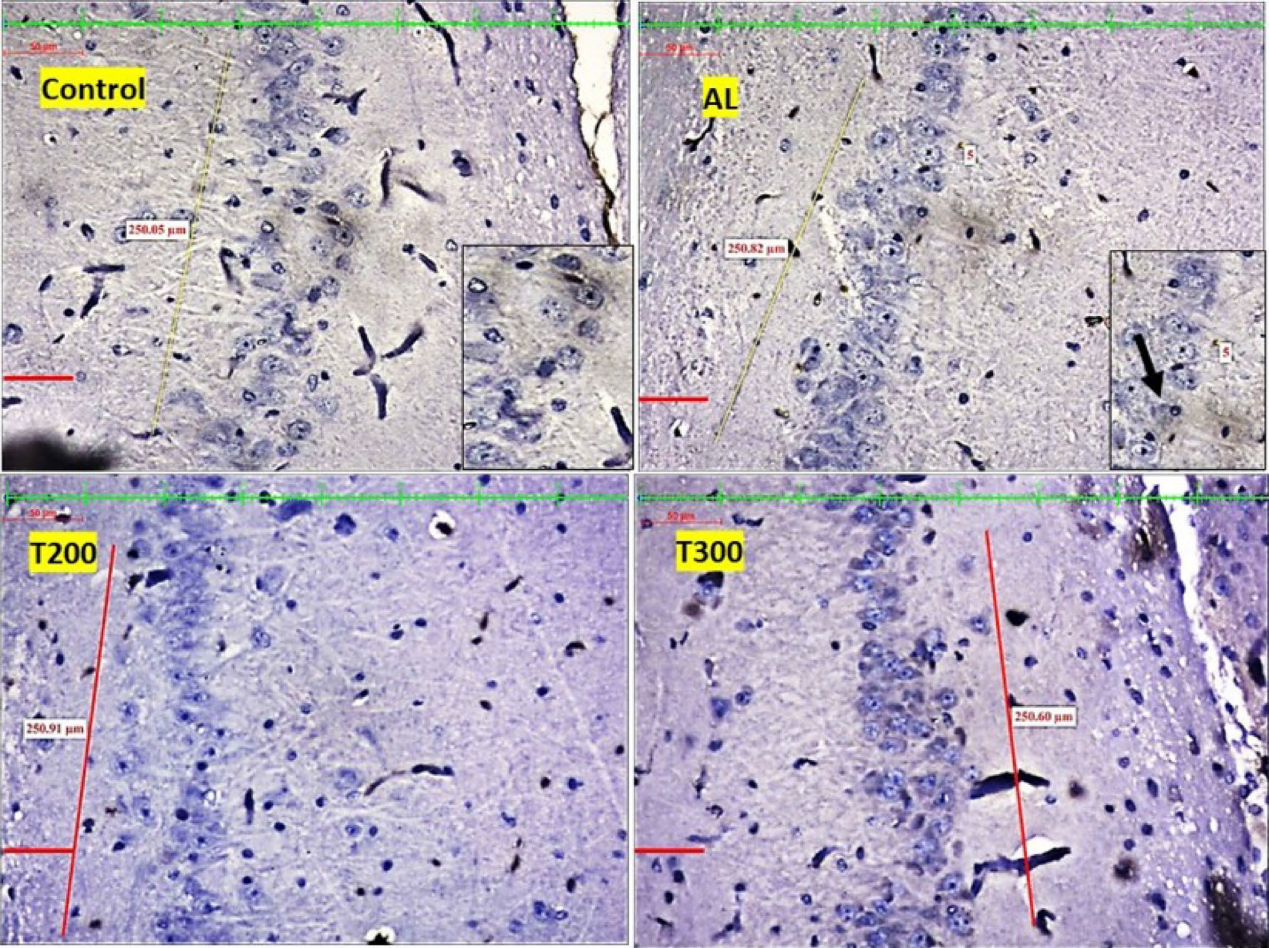
Photomicrograph of CA3 area of Hippocampus of rat brain tissue immunostained with anti- β-Amyloid antibody for β-Amyloid plaques (BAP). BAP appears like dark colored aggregation, usually circular extracellular deposits (black arrow); Scale bar-50µ

**Fig. 4:**
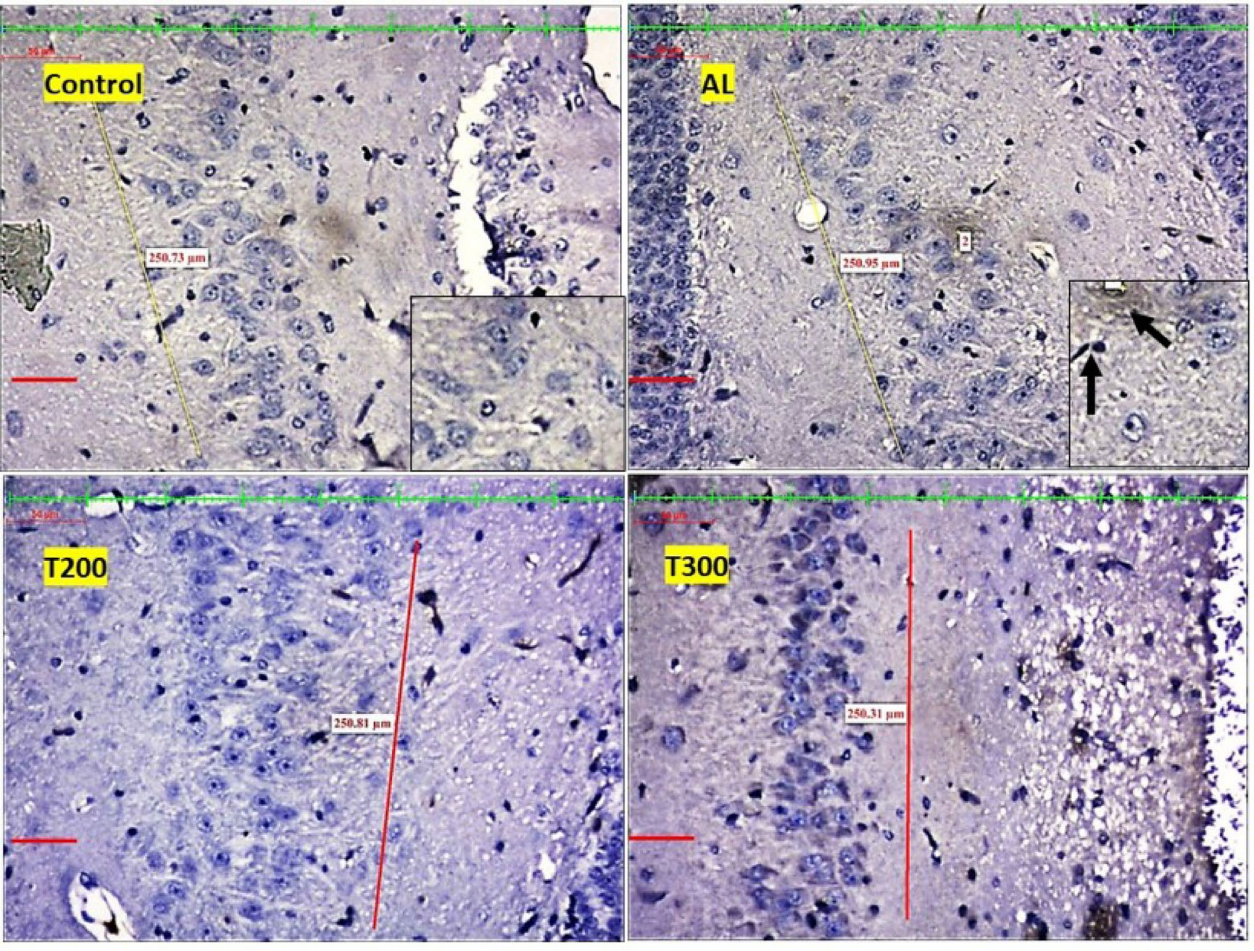
Photomicrograph of CA4 area of Hippocampus of rat brain tissue immunostained with anti- β-Amyloid antibody for β-Amyloid plaques (BAP). BAP appears like dark colored aggregation, usually circular extracellular deposits (black arrow); Scale bar-50µ

**Fig. 5:**
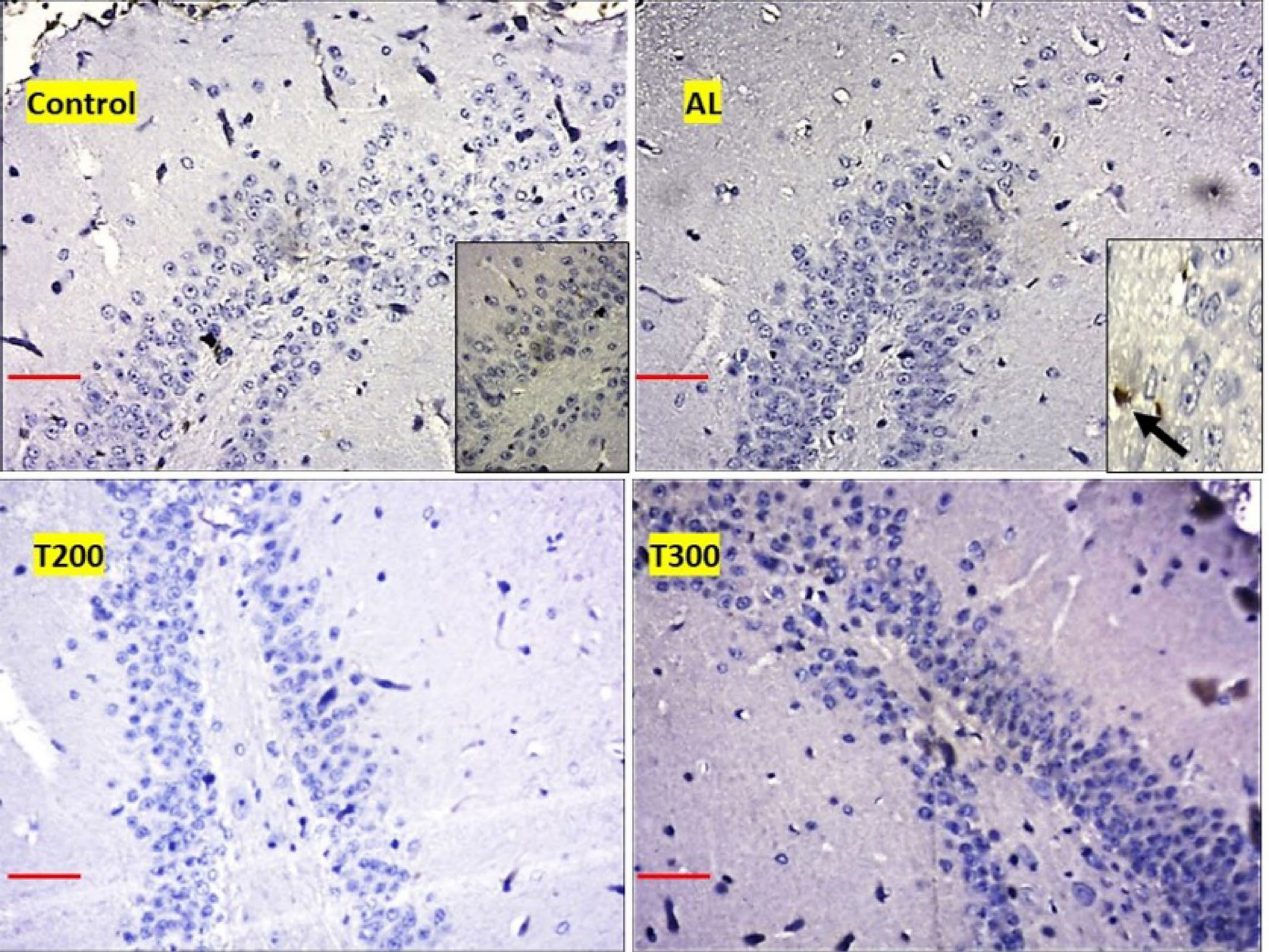
Photomicrograph of Dentate gyrus of rat’s brain tissue immunostained with anti- β-Amyloid antibody for β-Amyloid plaques (BAP). BAP appears like dark colored aggregation, usually circular extracellular deposits (black arrow); Scale bar-50µ

**Fig. 6:**
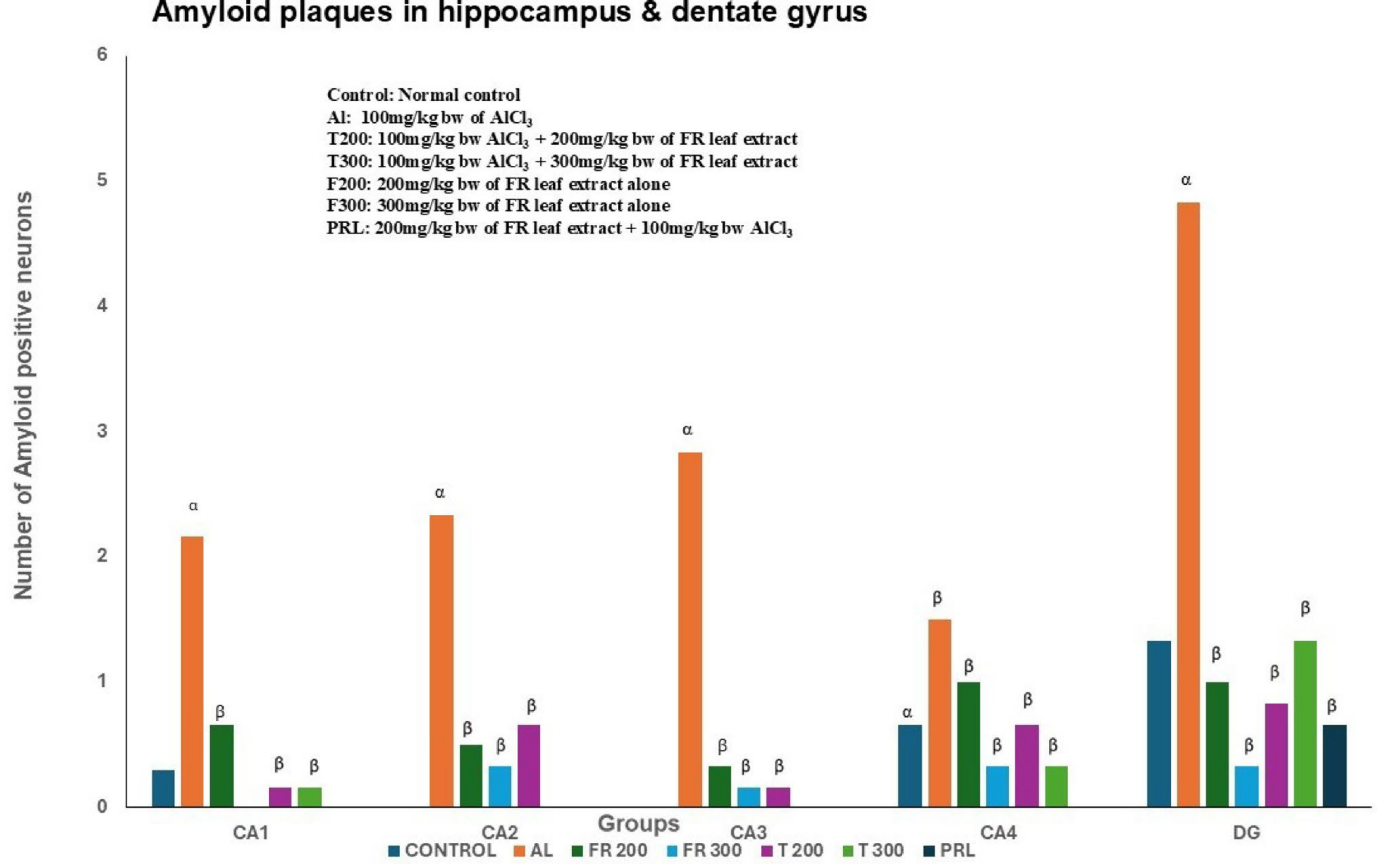
Number of Amyloid plaques in different areas of Hippocampus across different groups. α represents control vs. Al group, α – *p* < 0.001; β represents Al vs. rest of the groups, β - *p* < 0.001; Comparison by One-way ANOVA and Tukey multiple comparisons test; *n* = 6; Values are expressed as Mean ± SD

**Fig. 7:**
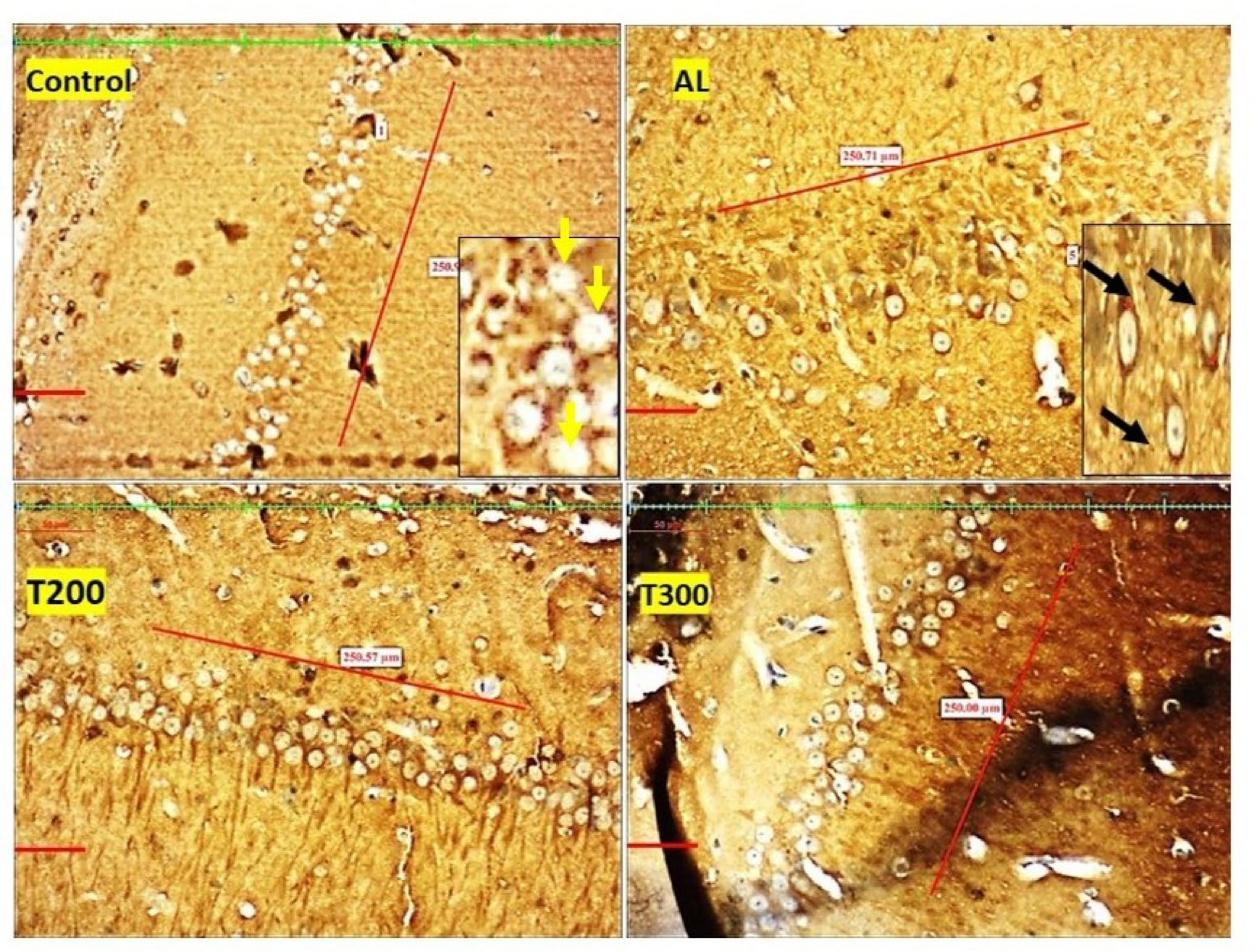
Photomicrograph of CA1 area of Hippocampus of rat brain tissue subjected to immunohistochemical examination of neurofibrillary tangles by anti-tau antibody. Black arrow- tau positive neurons; yellow arrow- tau negative neurons; Scale bar-50µ; Inset- higher magnification of the neurons

**Table 1:** Tabulation of amyloid plaques in different groups

Groups	CA1	CA2	CA3	CA4	DG
Al	2.16 ± 1.47	2.33 ± 0.51	2.83 ± 1.83	1.50 ± 0.83	4.83 ± 2.56
Control	0.33 ± 0.51	0.00 ± 0.00	0.00 ± 0.00	0.66 ± 0.81	1.33 ± 0.51
FR 200	0.66 ± 0.81	0.50 ± 0.54	0.33 ± 0.81	1.00 ± 1.26	1.00 ± 1.09
FR 300	0.00 ± 0.00	0.33 ± 0.51	0.16 ± 0.40	0.33 ± 0.51	0.33 ± 0.51
PRL	0.00 ± 0.00	0.00 ± 0.00	0.00 ± 0.00	0.00 ± 0.00	0.66 ± 0.81
T 200	0.16 ± 0.40	0.66 ± 1.21	0.16 ± 0.40	0.66 ± 0.81	0.83 ± 0.98
T 300	0.16 ± 0.40	0.00 ± 0.00	0.00 ± 0.00	0.33 ± 0.51	1.33 ± 1.03

Values are expressed as Mean ± Standard deviation; *n* = 6

**Table 2:** Tabulation of neurofibrillary tangles in different groups

Groups	CA1	CA2	CA3	CA4	DG
Al	5.33 ± 2.25	9.00 ± 1.09	8.33 ± 1.03	12.50 ± 3.93	21.33 ± 4.08
Control	2.00 ± 1.09	2.00 ± 0.89	1.50 ± 0.54	2.50 ± 2.07	16.66 ± 9.58
FR 200	2.00 ± 0.89	2.83 ± 1.47	2.66 ± 1.96	1.33 ± 0.81	11.50 ± 1.22
FR 300	2.33 ± 1.36	2.16 ± 0.75	2.16 ± 0.75	2.50 ± 1.37	11.16 ± 0.75
PRL	0.50 ± 0.54	1.00 ± 1.09	1.00 ± 1.09	1.00 ± 0.89	4.33 ± 3.14
T 200	1.00 ± 0.63	1.33 ± 0.81	2.16 ± 2.857	2.833 ± 2.63	12.50 ± 0.54
T 300	0.66 ± 0.81	0.50 ± 0.83	2.16 ± 1.47	1.50 ± 0.54	9.50 ± 3.83

Values are expressed as Mean ± Standard deviation; *n* = 6

**Table 3:** Tabulation of expression of AChE in different groups

Groups	AChE U/g tissue
Control	1.24 ± 0.46
Al	1.93 ± 0.62
FR 200	1.77 ± 0.3
FR 300	1.61 ± 0.32
T 200	1.59 ± 0.35
T 300	1.51 ± 0.24
PRL	1.38 ± 0.34

Values are expressed as mean ± SD; *n* = 6

### Neurofibrillary tangle formation (NFT): (Figures 7, 8, 9, 10 and 11; Table 2)

The neurofibrillary tangle was substantially (*p* < 0.001) higher in Al group vs. control rats, in all subregions of hippocampus. The NFT were found to be lesser in number in treatment groups T200 and T300, compared to Al groups, in all areas of hippocampus (Fig. 12). In dentate gyrus, Al group has shown increased number of NFT compared with control but not significantly increased. In T300 and prophylactic groups NFT has significantly (*p* < 0.001) declined in number in dentate gyrus compared with Al group. In drug-controlled groups (FR 200 and FR300) and T200 groups, NFT has moderately (*p* < 0.05) decreased in dentate gyrus compared to Al group.

**Fig. 8:**
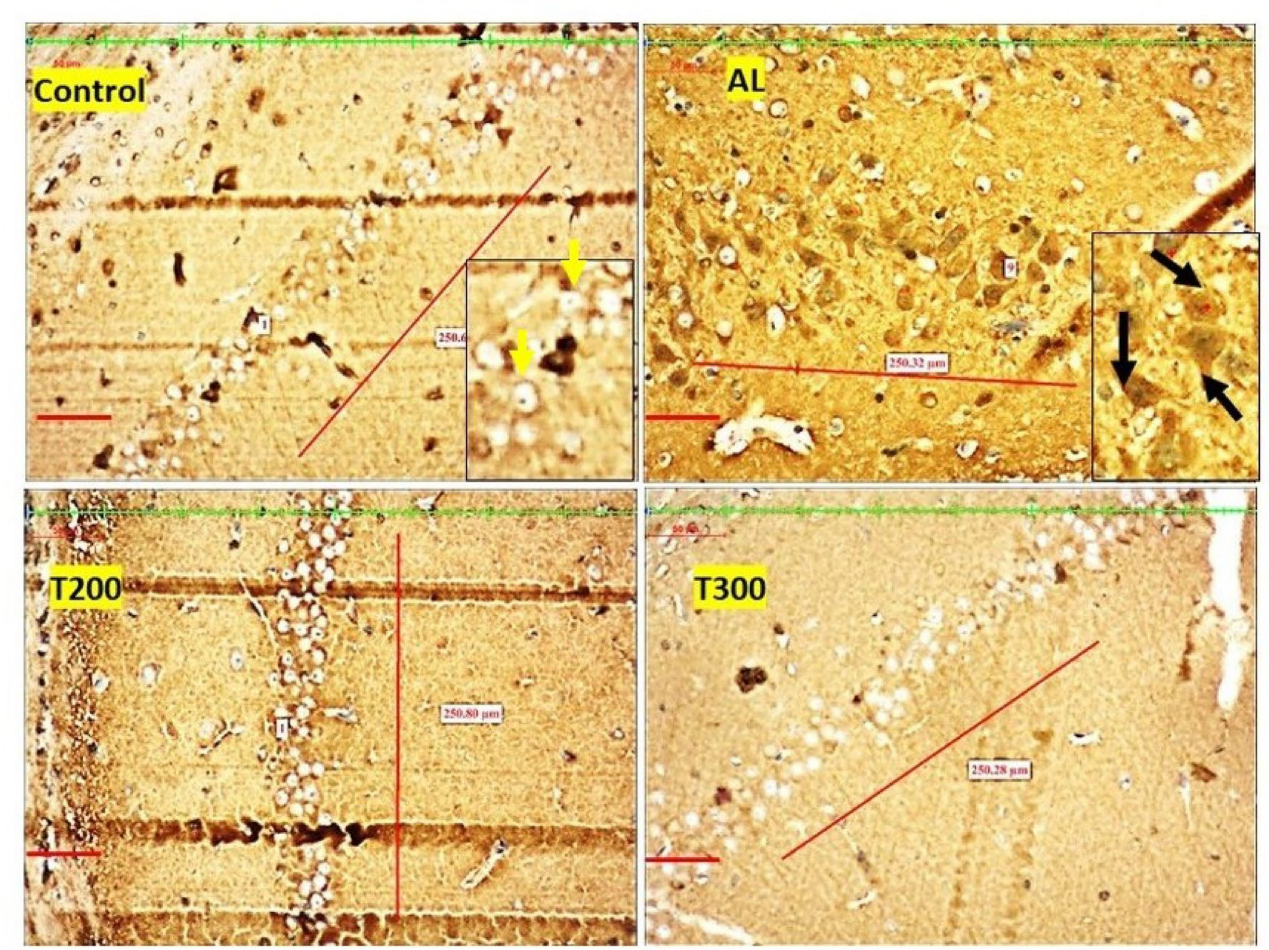
Photomicrograph of CA2 area of Hippocampus of rat brain tissue subjected to immunohistochemical examination of neurofibrillary tangles by anti-tau antibody. Black arrow- tau positive neurons; yellow arrow- tau negative neurons; Scale bar-50µ; Inset- higher magnification of the neurons

**Fig. 9:**
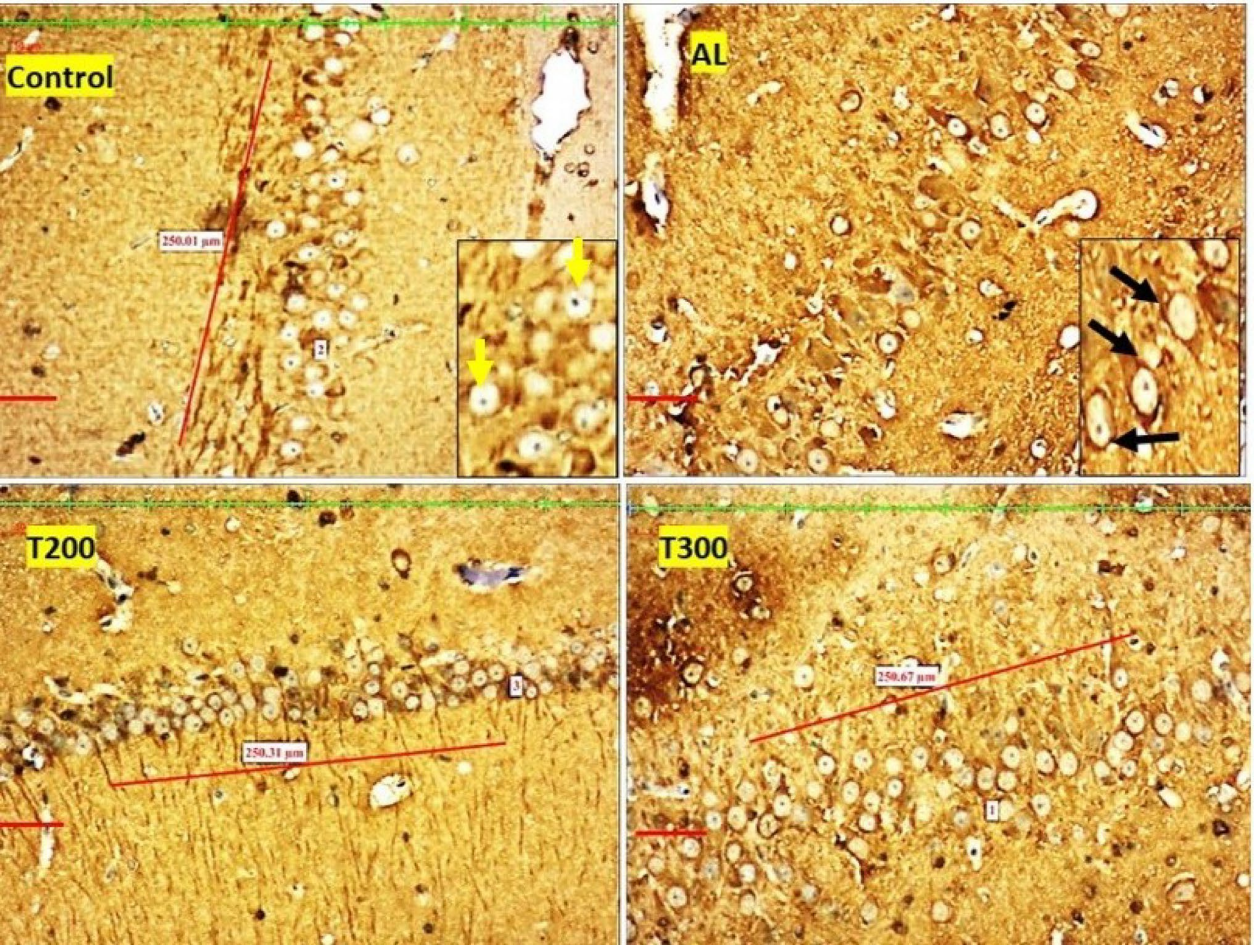
Photomicrograph of CA3 area of Hippocampus of rat brain tissue subjected to immunohistochemical examination of neurofibrillary tangles by anti-tau antibody. Black arrow- tau positive neurons; yellow arrow- tau negative neurons; Scale bar-50µ; Inset- higher magnification of the neurons

**Fig. 10:**
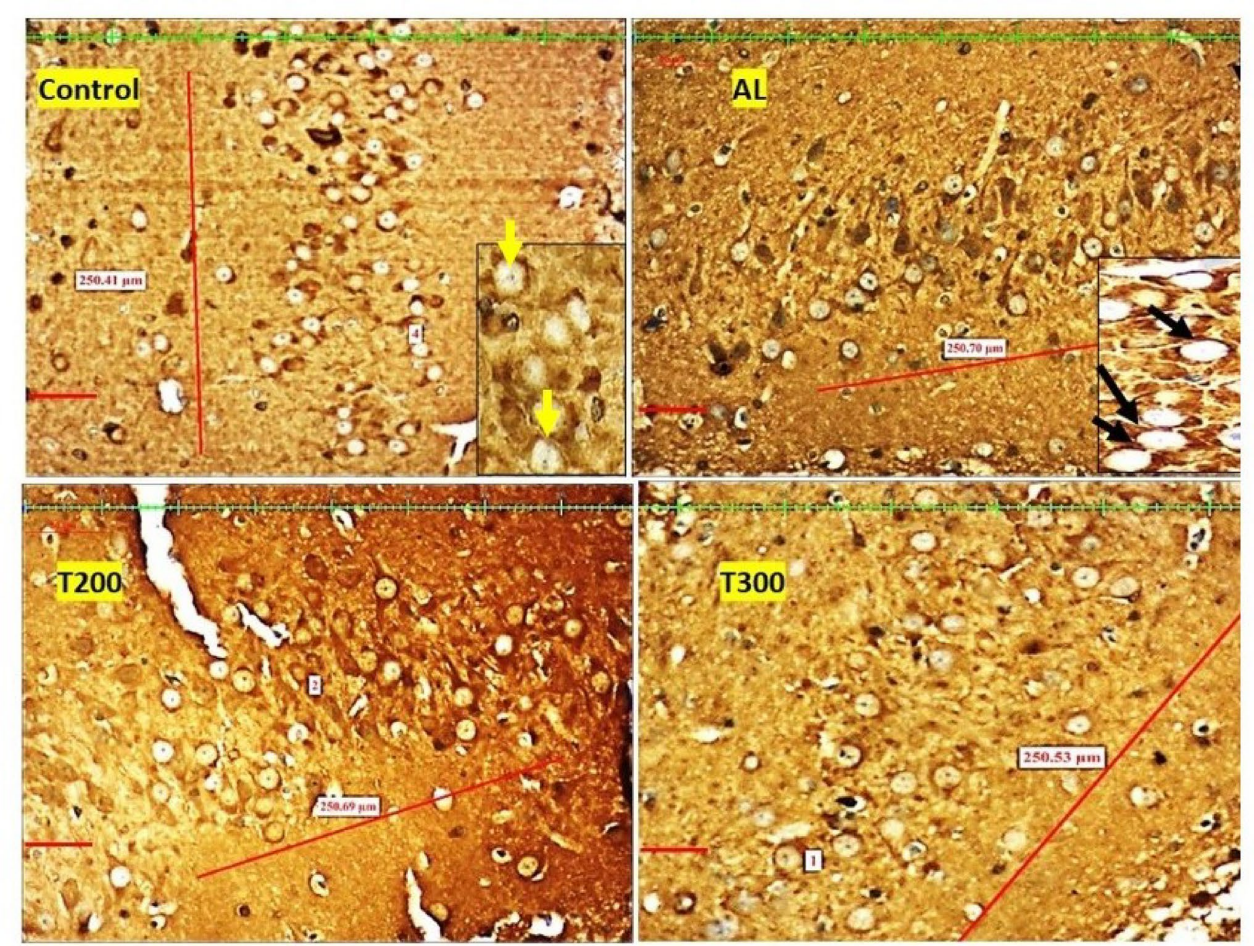
Photomicrograph of CA4 area of Hippocampus of rat brain tissue subjected to immunohistochemical examination of neurofibrillary tangles by anti-tau antibody. Black arrow- tau positive neurons; yellow arrow- tau negative neurons; Scale bar-50µ; Inset- higher magnification of the neurons

**Fig. 11:**
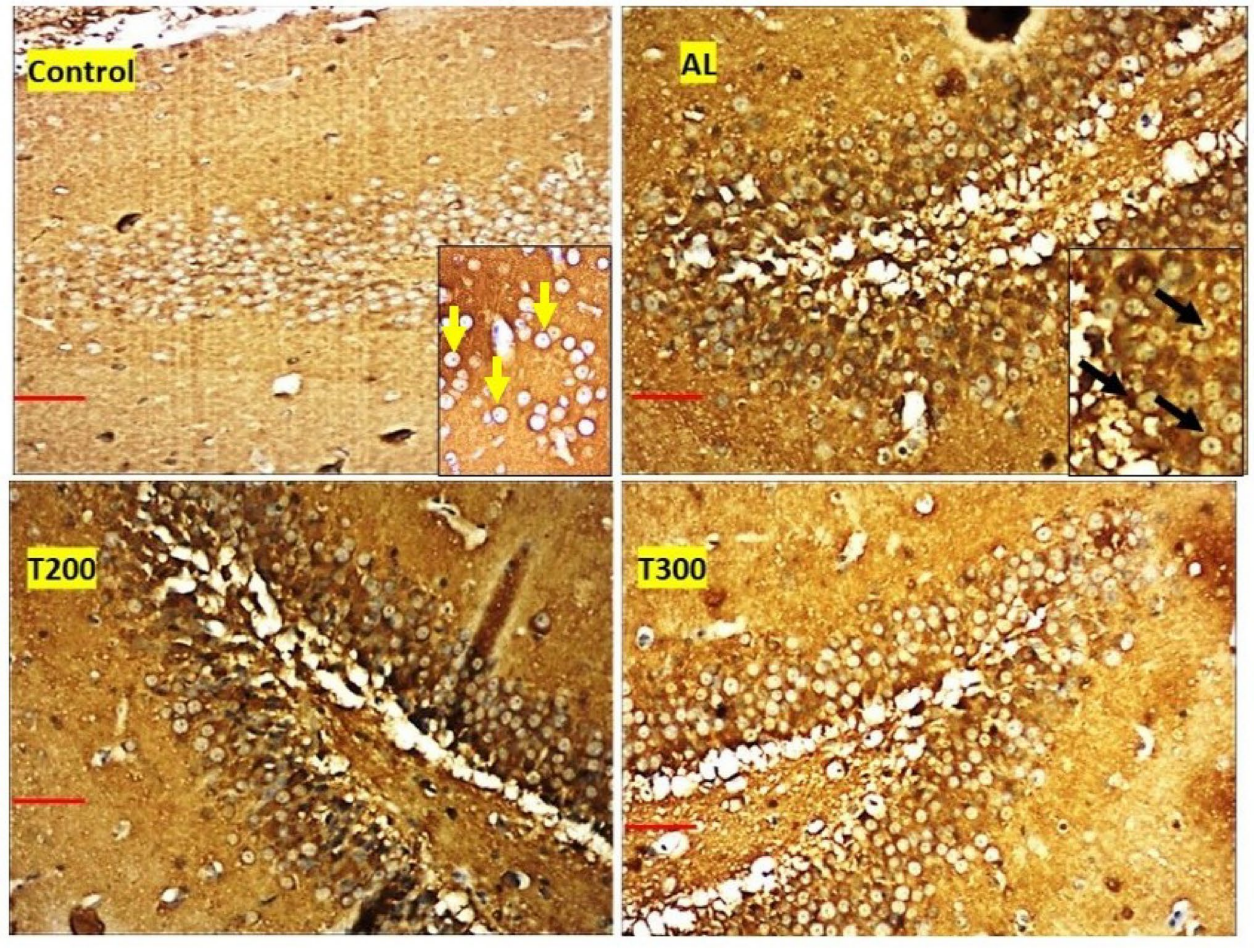
Photomicrograph of Dentate gyrus of rat brain tissue subjected to immunohistochemical examination of neurofibrillary tangles by anti-tau antibody. Black arrow- tau positive neurons; yellow arrow- tau negative neurons; Scale bar-50µ; Inset- higher magnification of the neurons

**Fig. 12:**
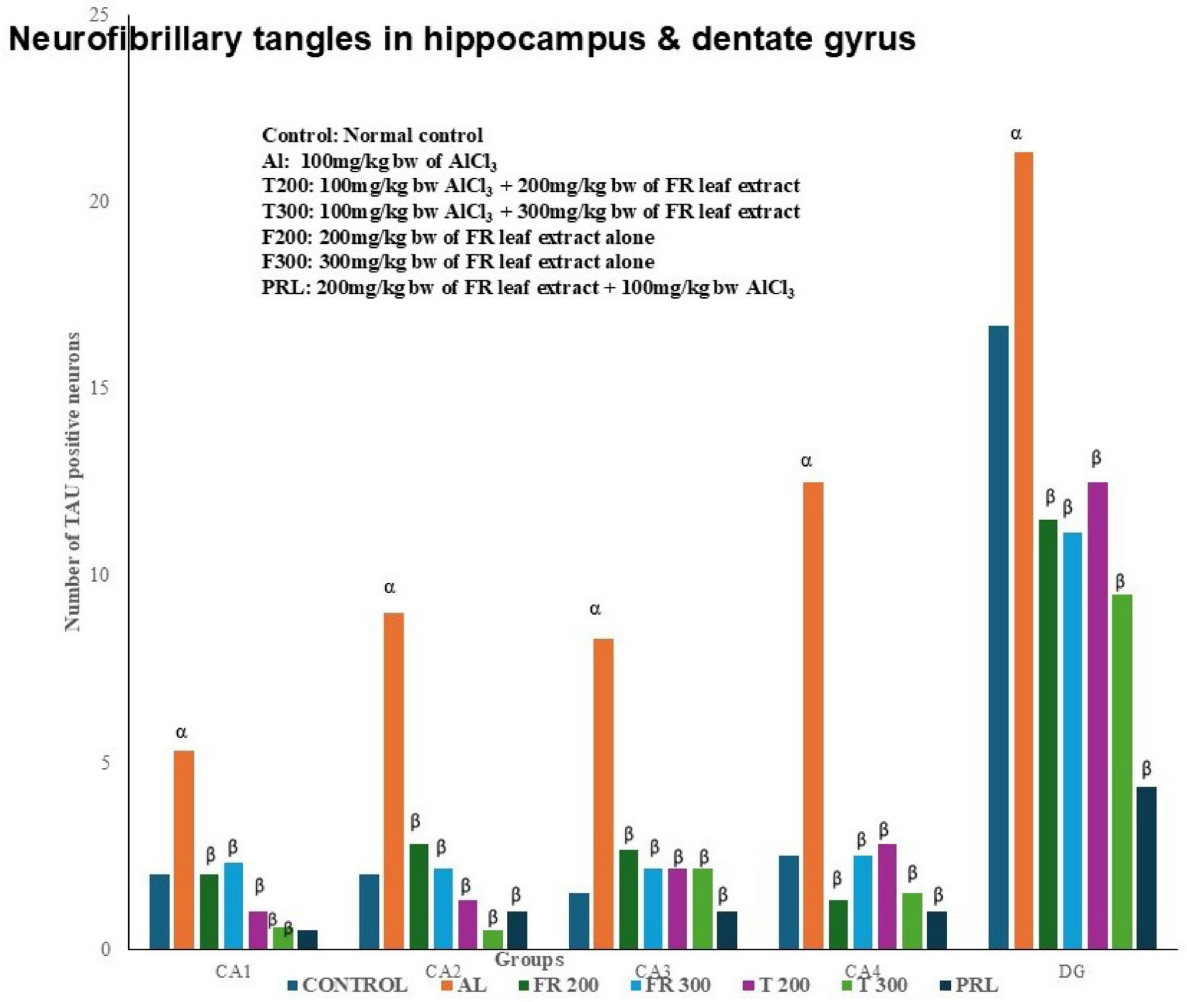
Number of Neurofibrillary tangles in different areas of Hippocampus across different groups. α represents control vs. Al group, α – *p* < 0.001; β represents Al vs. rest of the groups, β - *p* < 0.001; Comparison by One-way ANOVA and Tukey multiple comparisons test; *n* = 6; Values are expressed as Mean ± SD

**Fig. 13:**
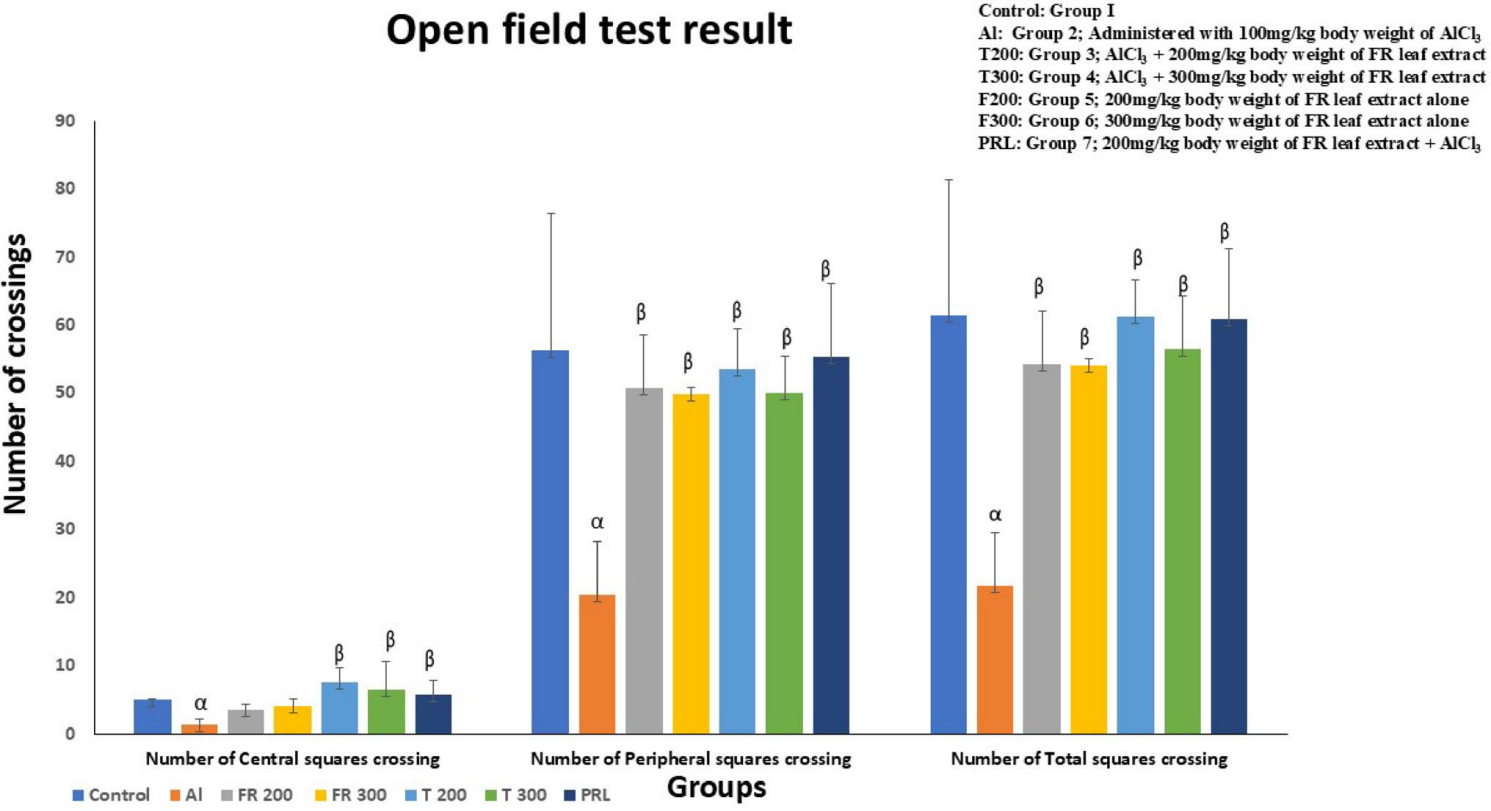
Comparison of number of crossings of rats (central, peripheral and total squares) between different groups. α represents control vs. Al group, α – *p* < 0.001 & αα – *p* < 0.05; β represents Al vs. rest of the groups, β - *p* < 0.001; Comparison by One-way ANOVA and Tukey multiple comparisons test; *n* = 6; Values are expressed as Mean ± SD, Error bar represents ± SD

**Fig. 14:**
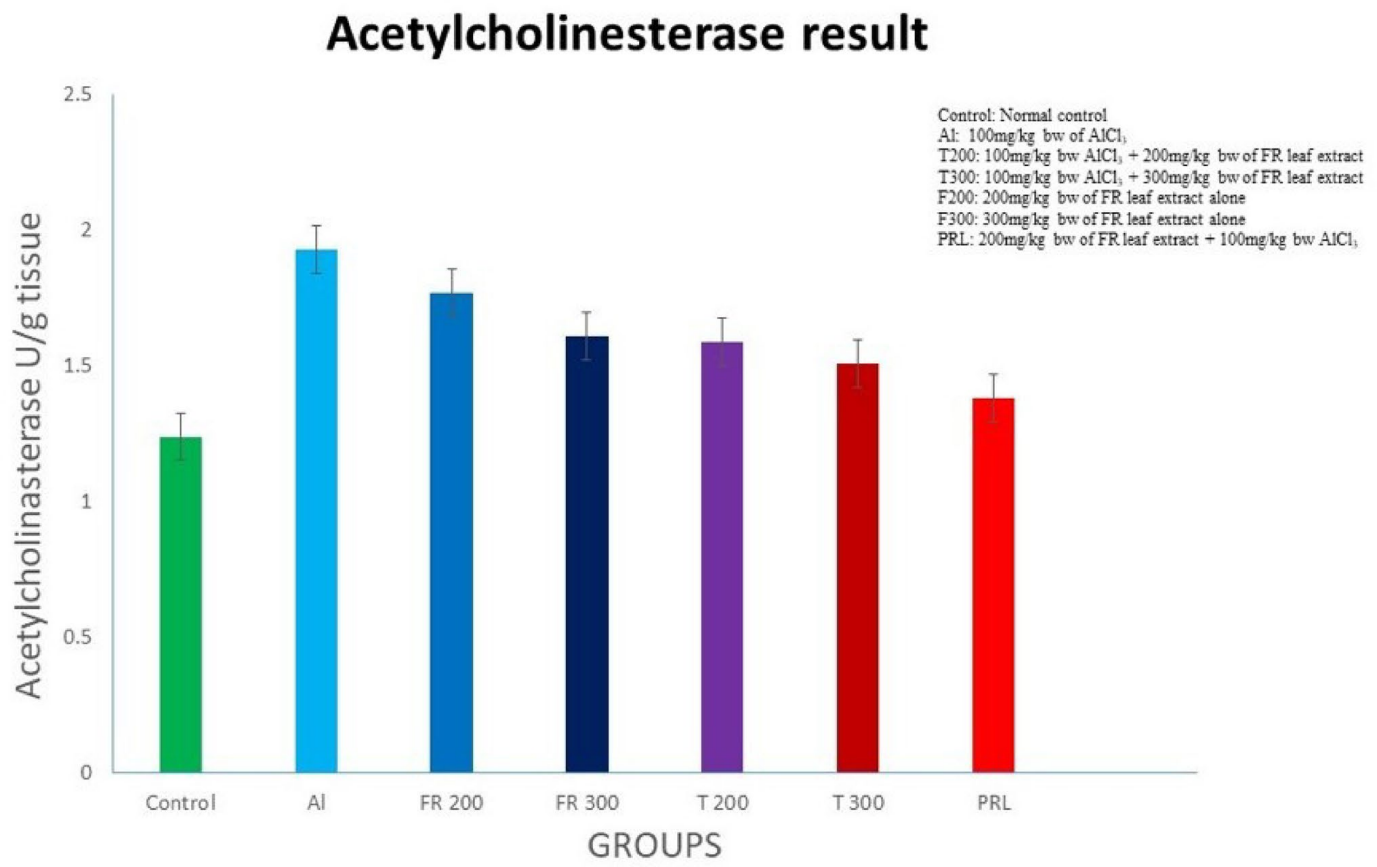
Acetylcholinesterase level in the brain tissue of different groups (*n* = 6). Values are expressed as mean ± SD. There was no significant difference in AChE level across the group; *n* = 6; Comparison by One-way ANOVA and Tukey multiple comparisons test; Values are expressed as mean ± SD

### Open field test

#### Central square crossings

In Al group, the number of central square crossing was moderately (*p* < 0.05) reduced compared to control group (Fig. 13). In FR treated groups (200 mg/kg and 300 mg/kg bw) there was a significant (*p* < 0.001) increase in the number of central square crossings compared to Al induced group indicating improvement in locomotor activity. The prophylactic group has also shown significant (*p* < 0.001) improvement in locomotor activity in the form of increase in the number of central squares.

#### Peripheral square crossings

In Al group, there is a significant (*p* < 0.01) reduction in the number of peripheral square crossings compared with control group (Fig. 13). In the treated groups (T200 and T300) a significant improvement (*p* < 0.001) in locomotor activity was noted in the form of increase in the number of peripheral square crossings when compared to that of Al group. In drug control as well as the prophylactic groups also, the number of peripheral square crossings has significantly increased (*p* < 0.001) compared to that of Al group.

#### Number of total square crossings

The total number of square crossings (central and peripheral) in Al group has shown significant reduction (*p* < 0.001) when compared to that of normal control group (Fig. 13). However, these crossings were significantly (*p* < 0.001) increased in treated groups, prophylactic groups, and only drug groups.

### Acetylcholinesterase (Fig. 14; Table 3)

In Al group the level of acetylcholinesterase has increased compared to control group but not to be found statistically significant (Fig. 14). In treated groups the level of AChE has decreased though statistically not significant, FR group and prophylactic group also displayed reduced AChE compared to Al group though there was no significant difference.

## Discussion

Aluminium induced neurotoxicity is a matter of major concern in recent years, as the environment is so much adulterated with Al that its exposure in various forms is unavoidable. Several studies have observed that substantially increased exposure to Al compounds can induce neurotoxicity (Khalil et al. 2020; Kumar et al. 2019; Bindhu et al. 2019; Rather et al. 2018; Prema et al. 2016). In our pilot study, we observed neurotoxicity in the form of degenerated neurons in prefrontal cortex at 100 mg/kg/bw AlCl_3_ by oral route for 45 days (Massand et al. 2022). Initially, the increased toxic metals, like aluminium will be observed only in the blood after fresh exposure and later as the exposure intensity increases, they will be transported from blood to other tissues or systems like CNS (Rosin 2009). Patients with neurodegenerative diseases (ND) when underwent chelation therapy showed improvement in their neurological symptoms and this recovery was corresponding to decreased aluminium level in their urine sample (Fulgenzi et al. 2015). Al enters the brain via three pathways: blood-brain barrier (BBB), respiration by olfactory tract and through cerebrospinal fluid (Brylinski et al. 2023). It accumulates in various areas of hippocampus, dentate gyrus and frontal cortex (Nampoothiri et al. 2015; Abutaweel et al. 2012). It can cross the blood-brain-barrier (BBB) and develop neurotoxic effect in the brain by lipid peroxidation which was evident in the form of distorted neuronal cell membrane and nucleus as reported by (Wang 2018; Kandimalla et al. 2016). Aluminium disturbs cellular function by inhibiting certain enzymes like hexokinase and glucose-6-phosphate dehydrogenase, thereby adversely affecting antioxidant defence mechanism and the morphological structure of the brain (Schifman & Luevano 2018). Mold and his co-authors observed that Al deposition in the brain is accompanied by neurofibrillary tangle formation and amyloid plaque in the brain (Mold et al. 2021). According to them Al inhibits the activity of protein phosphatase and affects phosphorylation or dephosphorylation of tau. In addition to changes in brain architecture, aluminium induced neurotoxicity also causes memory disturbance, weak motor activities or reduced coordination (Hoffman et al. 2014; Inan-Eroglu E. Ayaz, 2018).

### Effect of AlCl_3_ and FR on amyloid plaques and NFT

Aggregations of abnormal protein are one of the common findings amongst neurodegenerative disorders. It is opined that these protein aggregates cause mitochondrial dysfunction, that in turn induces apoptosis of neurons leading to neuronal death (Li et al. 2022). Collection of amyloid plaques and neurofibrillary tangles in the brain due to dysfunctional mitochondria lead to synaptic dysfunction associated with cognitive damage (Rai et al., 2020; Bergamini et al., 2004). Aggregation of α-synuclein in dopaminergic neurons of the substantia nigra is one such main histopathological marker in Parkinson’s disease (Vidovic et al. 2022). In Alzheimer’s disease insoluble aggregation of amyloid beta-peptide and Tau protein in neurofibrillary tangles are the common features (Gulisano et al. 2018; Nam et al. 2025). In multiple sclerosis hyperphosphorylated Tau aggregations in demyelinated areas are the common findings (Hoehne et al. 2025). Mitochondrial dysfunction due to amyloid beta protein aggregation has been observed both in patients and transgenic models of AD (Atlante & Valenti 2023; Hampel et al. 2021; Bell et al. 2021). In amyotrophic lateral sclerosis (ALS) mitochondrial dysfunction and apoptotic cell death due to mitochondrial SOD1 enzyme aggregation has been reported (Ying et al. 2024). The inflammatory mediators produced by the neuroglial cells, particularly microglia and other CNS macrophages play a major role in neuro-inflammation (Adamu, et al. 2024). Unhindered microglial activation can lead to neuronal damage due to abnormally increased production of pro-inflammatory mediators like TNFα (Kwon & Koh 2020), nitric oxide and interleukins, that eventually can generate oxidative stress and apoptotic cell death (Kwon & Koh 2020; Qin et al. 2023; Zhao et al. 2026).

In the present study, the neurofibrillary tangles and amyloid plaques were noted in different areas of hippocampus as well as in dentate gyrus in all groups in varied quantity. In AlCl_3_ group the neurofibrillary tangles and amyloid plaques were more in comparison to other group animals (Fig. 6). Furcila et al. (2019) reported that CA1 region of hippocampus was mostly affected by amyloid plaques and NFT while DG and CA3 regions of hippocampus are least affected by such protein aggregations. In our study, we have observed a greater number aggregation of Amyloid plaques in CA3 region and dentate gyrus in Al intoxicated group (Fig. 3). In CA4 region of hippocampus there was more of NFT compared to other areas of hippocampus in AlCl_3_ treated group of our study (Fig. 4). Treatment with FR leaf extract has reduced the number of Amyloid plaques and NFT in all areas of Hippocampus as well as in dentate gyrus (Fig. 5).

### Effect of AlCl_3_ and FR on locomotor activity and behaviour

Open field test is used to evaluate locomotor activity as well as emotional behaviours like anxiety and fearfulness (Rai et al. 2023; Gentsch et al. 1982). Decreased number of square crossings indicate declined locomotor activity and decreased grooming and rearing indicates emotional behaviour like anxiety and fear. Several studies have reported reduction in locomotor activity due to aluminium toxicity in rodents (Lal et al. 1993; Sharma et al. 2013; Yellamma et al. 2010). On the contrary Abd-Elhady et al. (2013) did not find significant difference in square crossings as well as grooming and rearing of animals. However, we observed a drop in the number of crossings in Al group animals. Increased deposition of amyloid plaques and NFT in the Al intoxicated animals of our study corresponded with decreased locomotor activity, which was evaluated through open field test. Al intoxicated rats displayed a significant decrease in the exploratory behaviour that was evident in the form of decreased number of square crossings. Reduced locomotor activity coinciding with declined AChE activity and memory disturbance is reported by Tair et al. 2016. However, in our study AChE activity did not show any significant difference in Al group animals and control animals (Fig. 14). Reduced locomotor activity in Al intoxicated rats of our study were improved by ethanolic extract of FR leaves (Fig. 13). This agrees with the previous studies on the positive effect of FR leaves on locomotor activity (Bhat et al. 2024; Bhangale & Acharya 2016). Petroleum ether extract of FR leaves improved the locomotor activity in Parkinsons disease induced rats (Bhangale & Acharya 2016b). Similarly, hydroalcoholic extract of FR leaves reduced anxiety in Swiss albino mice, as reported by Bhat et al. 2024.

### Effect of AlCl_3_ and FR on acetylcholinesterase (AChE)

The AChE is the serine hydrolase enzyme found in neuromuscular junction which convert neurotransmitter acetylcholine into acetic acid and choline leading to termination of nerve signal (Trang & Khandhar 2025). Any impairment in cholinergic transmission may increase the severity of AD (Ferreira-Vieira et al. 2016). The liberated choline will join acetyl-CoA and once again form acetylcholine that will be stored in synaptic vesicles of nerve fibres. The level of AChE enzyme increases in anxiety and depression (McCloskey et al. 2017). The Al interferes with the acetyl Co-A metabolism, which in turn may reduce the formation of acetylcholine (Bielarczyk et al. 1998). Earlier it was assumed that premature apoptosis of cholinergic neurons and build-up of neurofibrillary tangles forming hyper phosphorylated Tau are related to each other (Arriagada et al. 1992). Accordingly, the cognitive deficit in Alzheimer’s disease (AD) was attributed to cholinergic neuronal deficiency (Whitehouse et al. 1982). On the contrary, some studies showed that AChE inhibitors reduce the cognitive disturbance in AD and recommend it as an alternate therapeutic component (Noedberg 2006; Recanatini & Valenti 2004).

Acetylcholine plays significant role in memory function by increasing long term potential in the hippocampus. The impaired cholinergic transmission leads to cognitive impairment in different neurological disorders (Wang et al. 2009). In cognitive impairment and change in behaviour, the activity of AChE has been reported either increasing or decreasing (Prakash & Kumar 2009; Lakshmi et al. 2015). Cognitive dysfunction in the form of weakened learning ability and memory was substantially ameliorated by AChE inhibition through phenyl benzoxazole derivatives, as reported by (Srivastava et al., 2019). In another study inhibiting the activity of AChE through compound 6 g, a synthetic derivative of resveratrol, improved the cognitive deficit that was induced by scopolamine exposure (Tripathi et al., 2019).

In the present study, we observed elevated AChE level in AlCl_3_ induced group compared to control group after 45 days (Table 1) and this is also correlated with cognitive deficit (Fig. 13). Various studies on rat brain have observed similar result after Al exposure in rats (Firdaus et al. 2022; Haider et al. 2020). In a mouse model, long term exposure (84 days) to aluminium caused AChE enzyme activation and eventually lead to pathogenesis of AD (Li et al. 2023). Exposure to AlCl3 for 90days also showed increased AChE activity in rats, as stated by Tair et al. (2016). On the contrary, our study involving 25- and 45-days exposure to Al did not show any significant alteration in the AChE level (Fig. 14). Auti & Kulkarni (2019) also observed increased AChE activity in aluminium chloride treated rats in comparison to normal control rats. When Al was administered in lower dose (1.5 mg/kg/b.w.) for higher duration (60 days) (Martinez et al. 2017). AChE activity was found to be decreased. Whereas, in our study where 100 mg/kg/bw Al was administered for 45 days the activity of AChE increased. Ravi et al. (2000) observed a decrease in the level of AChE in hippocampus but in cerebral cortex the enzyme level was unaffected, when they administered 400 mg/kg AlCl_3_ for 15 days, in postnatal rats. In our study, when the Al intoxicated rats were treated with FR leaf extract the level of AChE has reduced, in treated group as well as in prophylactic group but this difference was statistically not significant (Fig. 14). The enzyme activity has slightly increased suggesting the positive effect of FR against Al induced anxiety development. FR leaf extract was effective on bronchospasm induced by acetylcholine in guinea pigs (Kapoor et al. 2011). The AChE inhibitory potential of Hairy root cultures of FR is confirmed by Gill & Siwach (2022). The AChE inhibitory activity of methanolic extract of stem bark of FR is observed by Siwach & Gill (2014) while screening the medicinal plants of India. Additionally, contemporary research has shown that fungal endophyte derived bioactive compounds have neuroprotective potential and these fungal endophytes are the microbes residing inside the plant tissue forming a symbiotic relation with the host plant (Prajapati et al. 2025). Some lead molecules derived from these endophytes have shown AChE inhibitory activity (Vig et al., 2022) against neurotoxicity. Therefore, further research on FR can be focused on exploring the potential of fungal endophyte associated with it, if any.

## Conclusion

The present study observed increased expression of amyloid plaques and NFT in the brain of rats those underwent aluminium exposure, which was coinciding with decreased locomotor activity confirming Al induced neurotoxicity. These adverse effects were mitigated by treatment with FR extract, revealing the ameliorative potential of FR leaves against Al induced neurotoxicity. Among the two different dosages 300 mg exhibited better effect against amyloid plaques and NFT, while 200 mg was found to be more effective for locomotor activity. However, acetyl choline esterase activity was not altered significantly in both Al exposure group as well as FR treated group animals. However, these adverse effects were mitigated by treatment with FR extract, revealing the ameliorative potential of FR leaves. Whereas acetyl choline esterase activity was not altered significantly in both Al exposure group as well as FR treated group animals. Our results suggest that both Al exposure and FR leaves do not modulate cholinergic pathway, though this needs to be verified through larger sample size and increased duration of dosages. In conclusion, our study observed positive neuroprotective effect of FR leaves on Al induced neurotoxicity. But these findings come with certain limitations like lack of cognitive tests, small sample size and single animal model. This leaves the avenue for future studies including in-depth exploration of mechanisms involved, adding more behavioural tests and biochemical estimations to strengthen the concept of therapeutic usage of FR leaves against neurological disorders.
